# The Effects of the Temperature and Termination(-O) on the Friction and Adhesion Properties of MXenes Using Molecular Dynamics Simulation

**DOI:** 10.3390/nano12050798

**Published:** 2022-02-26

**Authors:** Yao Deng, Yu Chen, Hanxu Liu, Xin Yan

**Affiliations:** 1 School of Mechanical Engineering and Automation, Beihang University, Beijing 100191, China; dengyao726@buaa.edu.cn (Y.D.); chenyu1238@buaa.edu.cn (Y.C.); liuhanxu@buaa.edu.cn (H.L.); 2 Advanced Manufacturing Center, Ningbo Institute of Technology, Beihang University, Ningbo 315100, China

**Keywords:** MXenes, functional groups, AFM, MD simulation, indentation, adhesion, friction

## Abstract

Two-dimensional transition metal carbides and nitrides (MXenes) are widely applied in the fields of electrochemistry, energy storage, electromagnetism, etc., due to their extremely excellent properties, including mechanical performance, thermal stability, photothermal conversion and abundant surface properties. Usually, the surfaces of the MXenes are terminated by –OH, –F, –O or other functional groups and these functional groups of MXenes are related surface properties and reported to affect the mechanical properties of MXenes. Thus, understanding the effects of surface terminal groups on the properties of MXenes is crucial for device fabrication as well as composite synthesis using MXenes. In this paper, using molecular dynamics (MD) simulation, we study the adhesion and friction properties of Ti_2_C and Ti_2_CO_2_, including the indentation strength, adhesion energy and dynamics of friction. Our indentation fracture simulation reveals that there are many unbroken bonds and large residual stresses due to the oxidation of oxygen atoms on the surface of Ti_2_CO_2_. By contrast, the cracks of Ti_2_C keep clean at all temperatures. In addition, we calculate the elastic constants of Ti_2_C and Ti_2_CO_2_ by the fitting force–displacement curves with elastic plate theory and demonstrate that the elastic module of Ti_2_CO_2_ is higher. Although the temperature had a significant effect on the indentation fracture process, it hardly influences maximum adhesion. The adhesion energies of Ti_2_C and Ti_2_CO_2_ were calculated to be 0.3 J/m^2^ and 0.5 J/m^2^ according to Maugis–Dugdale theory. In the friction simulation, the stick-slip atomic scale phenomenon is clearly observed. The friction force and roughness (*Ra*) of Ti_2_C and Ti_2_CO_2_ at different temperatures are analyzed. Our study provides a comprehensive insight into the mechanical behavior of nanoindentation and the surface properties of oxygen functionalized MXenes, and the results are beneficial for the further design of nanodevices and composites.

## 1. Introduction

A large family of two-dimensional transition metal carbides and nitrides (MXenes) has been extensively studied as a class of materials with superior properties since its discovery [1]. MXenes have a general chemical formula $M_{n+1}X_{n}T_{x}$, where M is an early transition metal (Ti, Zr, V, Cr, Mo, Sc, etc.); X is either carbon or nitrogen; T represents surface terminations of –O, –F, or –OH attached to MXenes surfaces during synthesis; and n is an integer, equal to 1, 2, or 3 [2]. MXenes are synthesized by selectively etching some atomic layers of their layered precursors and the majority of MXenes are derived from bulk ternary compounds known as MAX phases [3]. According to the existing studies, MXenes have great application potential in energy storage [4,5], sensing [6], sterilization [7], catalyzation [8,9,10], and electromagnetism [11], especially in the field of electrochemistry, such as supercapacitors [12] and Li-ion batteries [13].

In addition, MXenes have exceptional mechanical performance [14], thermal stability [15], photothermal conversion [16] and abundant surface properties [17]. The functional groups of MXenes have a strong affinity for polymers, which shows a great promise for synthesizing advanced composites [18]. The molecular groups on the surface of MXenes play an important role in controlling the overall stability and properties of MXenes. Thus, there is an urgent need to unveil the mysteries related to the properties determined by termination groups. However, there are obvious obstacles in experimental research to the revelation of the role of termination groups. Firstly, it is a significant challenge to synthesize MXenes with a single functional group [19]. In addition, the description of atomic-level processes occurs dynamically at the interface and observing atomic interactions within the interface is also a challenging task in the laboratory. Molecular dynamics simulation and *ab initial* calculation play an important role in the related studies. As described in the research of Mohammad et al. [17], the electronic and magnetic properties of MXenes without terminations differ greatly from those of their corresponding MAX-phase solids covered by functional groups (F, OH, and O). Among those functional groups, O-functionalized MXenes show unique properties compared to MXenes without terminations [17,20,21]. For instance, O-functionalized Ti_2_CT_x_ shows better thermodynamic stability than -OH and -F functionalized ones [22]. The critical role of O-surface termination on the tensile mechanical properties of thinnest 2D Ti_2_C MXene also had been studied [23].

Great efforts have been dedicated in the mechanical properties [21,24] and electronic applications [25,26] of Ti_2_C MXene. Nevertheless, its tribological and adhesion properties are also worthy of attention [27], due to the research that exhibited that MXenes can significantly improve the friction-reducing as well as anti-wear properties as an additive in lubricating oil or an enhancer in metal or polymer matrix composites [27]. MXenes can reach 50,000 times in a damping stability test, and density functional theory (DFT) calculation shows that MXenes can reduce the influence of external loads by dissipating energy, such as interlaminar bond interaction, interlaminar compression and slip/shear [28]. Moreover, the lubrication effect is determined by the tribological properties and adhesion of the materials [29]. Thus, from the point of view of tribological applications, adhesion properties should also be considered carefully.

The characterization of two-dimensional materials is more difficult than that of other macroscopic materials owing to the limited length-scale in the nano regime. Molecular dynamics (MD) simulations provide an efficient view of atom evolution as well as structure deformation. Borysiuk et al. [25] predicted the Young’s modulus of 597 GPa for bare Ti_2_C and 502 GPa for bare Ti_3_C_2_. Similarly, a multitude of atomistic indentation studies have been performed on graphene and the results provide an in-depth understanding of the experiment observation [30,31]. Due to the time-scale bottleneck of the MD calculation, some parameters, such as sliding velocity, are still difficult to match using an atomic force microscopy (AFM) experiment [32]. Some researchers adopt time-scale atomistic approaches to extend the timescale in the atomistic simulation on models not limited in friction or indentation [33,34,35,36]. It is undoubted that computational methods can provide guidance for experiments and obtain an insight into the microscopic mechanisms of MXenes.

According to the recent advances in the measurement of the elastic properties of MXenes, it is certain that indentation and friction can reveal more of the unknown properties of MXenes [37,38,39]. In this work, our objective is to study the effect of O-functional group and the temperature on the indentation and friction behavior using molecular dynamics simulation. Two sets of models mimicking AFM are built for indentation and friction. We carry out the indentation test and find the breaking point of Ti_2_C and Ti_2_CO_2_ at different temperature and evaluation the effect of the temperature on the breaking behavior under indentation. Additionally, we closely study the “jump in” and “jump out” phenomenon during approach, and withdraw and calculate the corresponding adhesion energy. Finally, we study the friction process and evaluate the friction force and surface roughness of Ti_2_C and Ti_2_CO_2_. Our study provides a fundamental insight into mechanical behaviors related to nanoindentation as well as the surface properties of oxygen-functionalized MXenes, which will benefit the further development of theoretical models, as well as nanodevice and composite designs.

## 2. Materials and Methods

**Figure 1:**
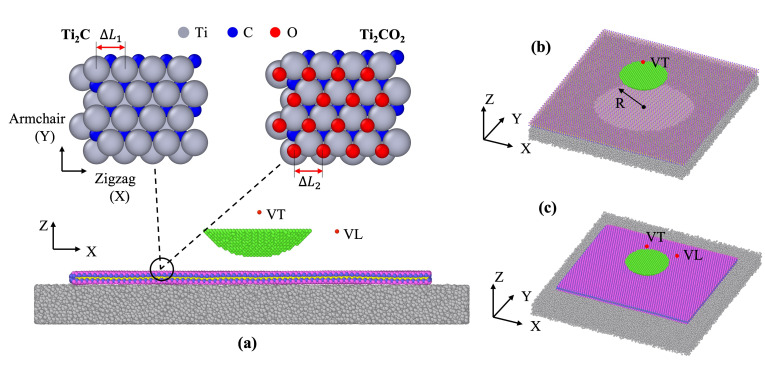
Atomistic models for the simulations. (**a**) The model setups, including amorphous substate, a paraboloidal tip and two virtual atoms (VT, VL). The two samples on the top are the atomistic structure of Ti_2_C and Ti_2_CO_2_. (**b**) Indentation model with a hole in an amorphous substate. (**c**) Friction model with a nonperiodic MXene flake.

In this work, two atomistic models were built for indentation and the friction simulations by mimicking AFM setups. Our atomistic model consisted of the sample (Ti_2_C or Ti_2_CO_2_), an amorphous Si substrate, a tip, and springs along the required directions. The amorphous Si substrate was created via a melting and quenching process and the size of the substrate was 24.3 nm × 24.7 nm × 2.0 nm, which contained 61,785 atoms. The atoms in the lowest 0.5 nm of the substrate were fixed during the entire simulation. The crystalline Si tip with a paraboloidal geometry was cut from a sphere (r_tip_ = 4 nm) that had a large curvature to ensure sufficient contact area with the MXenes. There were 4205 atoms in the tip, and it was set to be rigid in the simulation. Two virtual atoms with infinite mass, labeled VL and VT (Figure 1a), were employed to model the cantilever base and they were connected to the tip through a harmonic spring with a stiffness k_i_ (as shown in Figure 1a). Two types of MXenes atomic structure are shown in Figure 1a too. The MXene flake was parallel to the x-y plane, and the out-of-plane direction was along *z*-axis.

Due to the hexagonal arrangement of its atomic structure, MXenes also have two inherent material orientations, similar to graphene, and they are called armchair and zigzag (as shown in Figure 1a). This material orientation may introduce anisotropic feature of the MXenes.

The indentation model depicted in Figure 1b contained only one necessary virtual atom VT, which was connected to the tip through a spring with stiffness of $k_{1}$ = 200 $N\;m^{-1}$. In addition, a hole with radius R was created in the center of the Si substrate. In this model, the MXene flake was with the same dimension as the amorphous Si substrate, and we applied a periodic boundary condition in the X and Y directions.

As shown in Figure 1c, the friction model consisted of two virtual atoms (VL and VT) controlling the horizontal motion of the tip and applying normal loads, respectively. In the vertical direction, the virtual spring between VT and the tip had the stiffness coefficient $k_{z}$ and, by adjusting the distance between the tip and VT, we could control the applied normal load. In horizontal direction, the spring connected to VL makes the tip to move, and the speed of VL is constant of $v_{f}=3\;m\;s^{-1}$. We would like to remark that this speed may be much faster than the experimental settings, and to give a realistic evaluation of speed effect (which is not included in current study), time-scaling atomistic simulation approaches could be adopted [35]. In the friction model, the size of MXene flakes we used were 20.3 nm × 17.0 nm and it contained around 88,000 atoms.

Owing to the diversity of atoms present in these two complex models, multiple potentials were applied to describe the dissimilar atomistic interactions in our models. Firstly, the ReaxFF [40] interatomic potential was used within the atoms in MXenes. This potential has been widely used in the study of MXenes and appears to provide a reasonable description of their mechanical properties [41] and thermo properties [42]. Then, the Lennard–Jones (LJ) potential [43,44] was acted for MXenes with the tip and substrate calculation. The interactions in the Si substrate and tip were described using the sw potentials [45]. Before implementing the loads, the MXene flake was put on the amorphous substrate and equilibrated using NVE ensemble at the preset temperature for 100 ps. Additionally, during loading, we applied NVE all through the process. Our MD simulations were performed using Largescale Atomic/Molecular Massively Parallel Simulation (LAMMPS) [46] and atomic imaging was performed in OVITO [47].

## 3. Results and Discussion

### 3.1. Indentation Simulation

**Figure 2:**
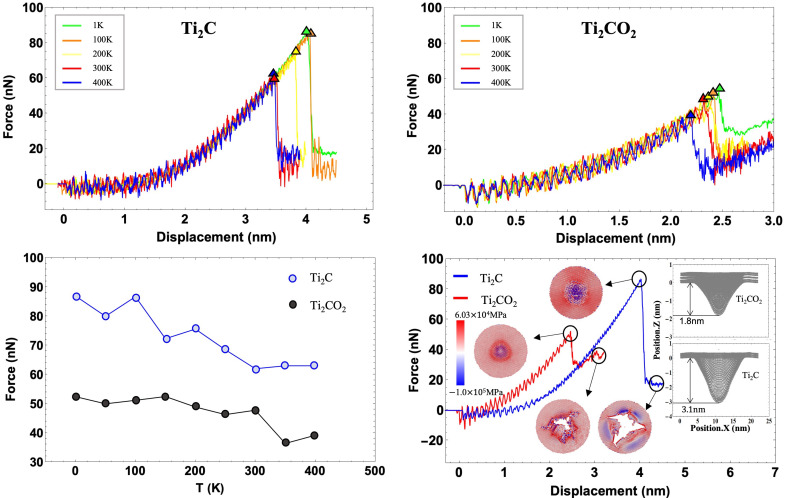
(**a**,**b**) Force–displacement curves of Ti_2_C and Ti_2_CO_2_ at different temperatures; (**c**) The breaking force of Ti_2_C and Ti_2_CO_2_ at different temperatures; (**d**) force–displacement curves of Ti_2_C and Ti_2_CO_2_ at 1 K. The snapshots correspond to the stress analysis, which are colored by the stress in the Z direction. The insets illustrate the deformation of the atomistic models of Ti_2_C and Ti_2_CO_2_ at the breaking points on the force–displacement curves.

The first sequence of simulations was the nanoindentation test of Ti_2_C and Ti_2_CO_2_ in the temperature range from 1 K to 400 K. The tip was pressed at a constant speed $v_{d}=10\;m\;s^{-1}$ until the surface of the MXene flake broke. The force–displacement curves of the indentation test on Ti_2_C and Ti_2_CO_2_ are shown in Figure 2a,b. Overall, we observed that, as the tip is pressed down, the force gradually increases until the breaking point, after which the force drops suddenly. Moreover, all the curves overlap completely before breaking occurs. When the fracture occurs, as the temperature increases, both the fracture force and the displacement corresponding to the breaking point decrease. The variation of fracture force versus the temperature of Ti_2_C and Ti_2_CO_2_ are shown in Figure 2c. In general, with the increase in the temperature, the breaking force reduces for both Ti_2_C and Ti_2_CO_2_. Scrutiny reveals that the breaking force of Ti_2_C is more sensitive to temperatures below room temperature at 300 K, but when the temperature increases to above room temperature, the breaking force of Ti_2_CO_2_ become more sensitive to the temperature change.

To have a direct comparison between Ti_2_C and Ti_2_CO_2_, the force–displacement curves of Ti_2_C and Ti_2_CO_2_ at 1 K are shown together in Figure 2d. As can be seen, the curve of Ti_2_CO_2_ is steeper than that of Ti_2_C before the fracture occurs, which means the stiffness of Ti_2_CO_2_ is higher. In addition, we exhibit four snapshots of atoms colored by the stress in Z direction corresponding to the circled points on the force–displacement curves. At the summit of the force, stress concentration happens in the central region of Ti_2_C and Ti_2_CO_2_ (the top two snapshots). After fracture, Ti_2_CO_2_ has a greater residual stress on the atoms near the crack (the bottom two snapshots). The insets in Figure 2d exhibits the maximum deflection before the fracture of Ti_2_C and Ti_2_CO_2_. In contrast to Ti_2_C, the fracture of Ti_2_CO_2_ occurs at a smaller deflection of 1.8 nm and a reduced breaking force of 52 nN.

**Figure 3:**
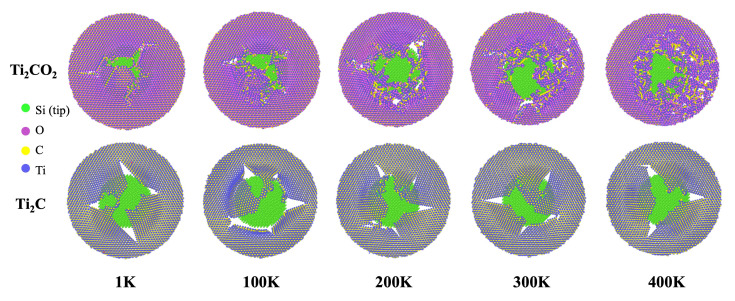
Fracture morphology of Ti_2_C and Ti_2_CO_2_ with temperature changes from 1 K to 400 K.

We carried out a further investigation of the breaking morphology of Ti_2_C and Ti_2_CO_2_. The crack snapshots of Ti_2_C and Ti_2_CO_2_ samples at different temperatures are shown in Figure 3. On the one hand, we noticed that both cracks of Ti_2_C and Ti_2_CO_2_ initiate and grow along zigzag directions, and this can be clearly observed in the snapshots at 1 K. Our results are in good agreement with the published MD simulation results, which indicate that MXenes tend to fracture along zigzag directions under tensile loading [48]. On the other hand, with the increase in the temperature, the crack edge of Ti_2_CO_2_ becomes blurred, while for Ti_2_C, the crack edge remains clear through all the temperatures. In addition to the main crack, small cracks appear around for Ti_2_CO_2_. In that sense, the temperature has a stronger influence on the morphology of Ti_2_CO_2_. After the breaking occurs, there are many residual bonds in Ti_2_CO_2_ leading to the formation of a blurred crack edge, which is also the main reason for the large residual stress in Ti_2_CO_2_ shown in the force–displacement curves in Figure 2d. In addition to the unbroken Ti–O bond, there are many new bonds formed due to oxidation.

In addition to the breaking strength, the adhesion of MXenes is another critical surface characteristic worth exploring. In this paper, we carried out a set of simulations to study the adhesion properties of Ti_2_C and Ti_2_CO_2_ flakes at different temperatures. The simulation process involved tip approaching and withdrawing. All the tests were performed with the tip moving at a constant velocity of 10 $m\;s^{-1}$.

**Figure 4:**
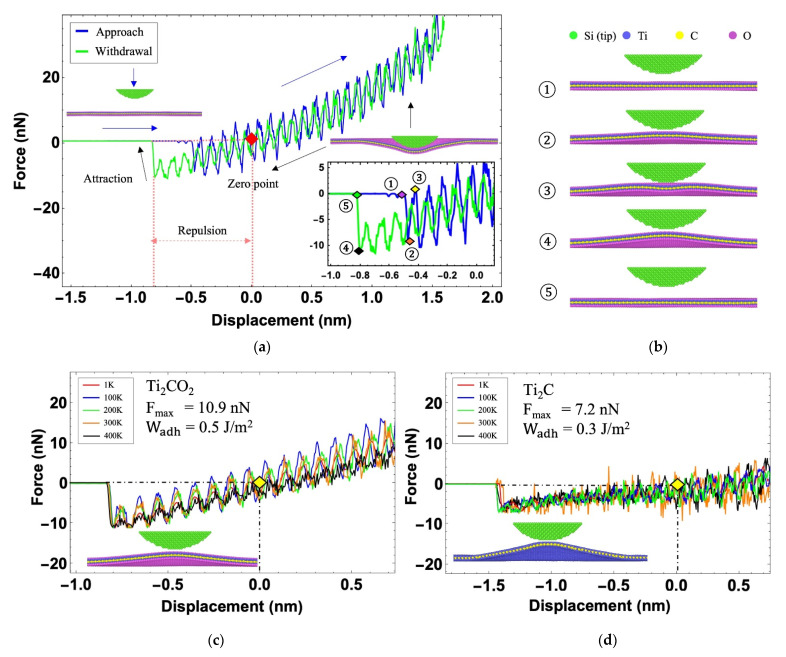
(**a**) Approach and withdrawal process for Ti_2_CO_2_ at 300 K. Two snapshots show the initial position of approach and withdrawal. The inset shows the key adhesion stages. (**b**) Snapshots of the “jump in” and “jump out” phenomenon. (**c**) Withdrawal curves for Ti_2_CO_2_ flake at different temperatures. (**d**) Withdrawal curves for Ti_2_C flake at different temperatures.

A typical force–displacement response of Ti_2_CO_2_ measured at 300 K is delineated in Figure 4a. In the atomistic model, a virtual atom with a spring was adopted to mimic the cantilever beam. Before the tip becomes close to the Ti_2_CO_2_ sample, the force remains at zero and the tip suspends away from the sample (the left snapshot in Figure 4a). As the tip approaches (indicated by the blue curve and arrows in Figure 4a), the force on the tip increases. When the tip moves down and reaches the depth of 1.5 nm below the sample, it starts to move back. During the tip moving back, the force applied on the tip reduces and, at some point, it recovers to zero (light green curve in Figure 4a). The inset is the zoomed-in view of the curve corresponding to the “jump in” and “jump out” process. The circled numbers correspond to the snapshots in Figure 4b.

In Figure 4b, the differences in height between the tip and the sample surface in snapshots 1 and 2 or 4 and 5 clearly reflect the occurrence and disappearance of the adhesion phenomenon. As the tip moves towards the Ti_2_CO_2_ flake, “jump in” occurs before tip reaches zero point (the initial height of the Ti_2_CO_2_ sample is set as zero point and marked with a red diamond in Figure 4a). Together with the “jump in” phenomenon, the adhesion force increases significantly (indicated by the orange diamond point in the inset) as well as the bulge of the sample in Figure 4b (snapshot 2). We noticed that the surface shapes of the structure in snapshots 2 and 3 in Figure 4b are slightly different. There is a slight dent in the middle of sample surface in snapshot 3. This is the result of continuous competition between positive pressure and adhesion force. At the end of competition, the tip arrives at the zero point (Figure 4a) and the adhesion force gradually decreases to 0. In the withdrawal process, the Ti_2_CO_2_ flake is attached to the tip the entire time until tip passes zero point and “jump out” with sample. At the snapshot 4 in Figure 4b, the adhesion force reaches its maximum in the whole simulation (the black diamond in the inset of Figure 4a). The maximum adhesion force measured at the end of separation stage can be used to calculate the adhesion energy between the tip and the MXene flake.

Generally, the adhesion energy $W_{adh}\left[J/m^{2}\right]$ is used to describe the energy required (for unit area) to separate two perfect planes from equilibrium contact to infinity [49]. Here, Maugis–Dugdale theory [50] is applied to extract the adhesion energy as

(1) $$W_{adh}=\frac{F_{max}}{\lambda \pi r},$$

where $F_{max}$ is the maximum adhesion force during the indentation process, r is the radius of the tip, and λ is an effective coefficient that ranges between 1.5–2. The value of λ depends on the contact pair (λ=1.602 for monolayer Ti_2_CT_x_ [51], λ=2 for Ti_2_C [49]). Equation (1) assumes that the work of adhesion is measured by perfectly smooth tip. Here, the effect of roughness on adhesion energy is not considered.

Figure 4c,d illustrate the force–displacement curves during the withdrawal process of Ti_2_C and Ti_2_CO_2_ at different temperatures. As the tip moves away from the surface of the MXene sample, the force decreases to zero when the sample recovers to the original place (yellow diamond in Figure 4c,d). When the tip moves up continuously, the MXene sample sticks to the tip, which leads to an increase in the absolute value of the force (but opposite sign) until the sample and the tip separate from each other and the force drops rapidly to zero. We noticed that the maximum adhesion force of the same material at different temperatures is almost the same. In another words, the temperature does not affect the maximum adhesion force and the adhesion energy based on our simulation. According to Equation (1), the adhesion energies of Ti_2_CO_2_ and Ti_2_C are 0.5 $J/m^{2}$ and 0.3 $J/m^{2}$. Furthermore, the maximum separation distance between the sample and the substrate is 1.4 nm and it is larger than the value of Ti_2_CO_2_ (0.8 nm, inset in Figure 4c,d), indicating there are stiffness differences between them. In summary, the surface functional group –O plays the role of enhancing the adhesion and stiffness of MXenes.

**Figure 5:**
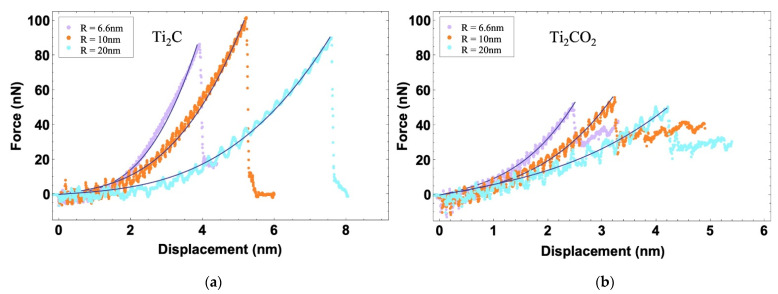
Force–displacement curves of (**a**) Ti_2_C and (**b**) Ti_2_CO_2_ of indentation using models with different hole radii (R = 6.6 nm, 10 nm, 20 nm).

The experimental study proposed by Lee [30] has shown that the ratio of tip radius to hole radius is related to the force–displacement response. In our work, we changed the ratio by modifying the radius of the hole to study the influence of the model on the force–displacement curve. Therefore, we constructed two additional models with the holes of 10 nm and 20 nm, and both were larger than the previous hole (R = 6.6 nm). The force–displacement curves of Ti_2_C and Ti_2_CO_2_ at 1 K are shown in Figure 5. Models of all sizes follow the same trend: as the tip is pressed down, the force increases until the MXene flake breaks. Lee et al. [30] proposed an elastic plate theory that is wildly applied for analysis the indentation process [52]. The formula is as follows:

(2) $$F=\sigma_{0}^{2D}\pi \delta +E^{2D}q^{3}\frac{\delta^{3}}{R^{2}},$$

where F is the normal force and δ is the deflection in the bending process. R represents the radius of the hole in the substrate. $\sigma_{0}^{2D}$ is the pretension stress in the membrane, $E^{2D}$ is the 2D elastic modulus related to the Young’s modulus E and the film thickness t: $E=E^{2D}/t$. $E^{2D}$ and $\sigma_{0}^{2D}$ are the unknown parameters and need to be determined via fitting the force–displacement curves. The thickness of MXenes used in this study were 0.56 nm for Ti_2_C and 0.60 nm for Ti_2_CO_2_. q is a dimensionless constant, which related to Poisson’s ratio v and is calculated by the following equation: $q=1/\left(1.049-0.15v-0.16v^{2}\right)$. In this study, v is taken from the DFT calculation [53] and the values of v were 0.23 and 0.32 for Ti_2_C and Ti_2_CO_2_, respectively.

Following Equation (2), we plotted the theoretical solution in Figure 5 (black solid curves). Our simulation results match the theoretical prediction very well. The force–displacement behavior presents a nonlinear elastic stress–strain response. We note that the fracture forces of the three models are close to each other, but the inclinations of the curves are evidently different. The flake breaks at a larger deflection (the blue curve) in the test with a larger hole in the substrate (R = 20 nm). The intrinsic strength of the MXene flake could be abstracted from the break point on the force–displacement plots in Figure 5 and calculated by a function as follows [30]:

(3) $$\sigma_{max}^{2D}=\sqrt{\frac{F_{f}E^{2D}}{4\pi r}},$$

where $\sigma_{max}^{2D}$ is the maximum stress, $F_{f}$ is the fracture force and r is the radius of the tip. Equation (3) illustrates the relationship among the maximum stress, tip radius, and fracture force. Based on the force–displacement data of loading the MXene flake to the breaking point, as well as the fracture force and the elastic theory of two-dimensional materials, the elastic constants of Ti_2_C and Ti_2_CO_2_ in the three models with different hole radii are calculated and listed in Table 1.

The influence of the substrate hole radius on the elastic stage of deformation is evident, but on breaking force and $\sigma_{max}^{2D}$, it is relatively small. From the study proposed by Lee et al. [30], we know that the breaking force is mainly a function of the tip radius and shows no dependence on the size of the hole, because of the extreme stress concentration in the center of the flake. The radius of the tip remains constant (r = 4 nm) in our models, and this is the main reason for the tiny difference in $\sigma_{max}^{2D}$. In contrast to Ti_2_C, the calculated Young’s modulus E of Ti_2_CO_2_ is higher, but the average intrinsic strength $\sigma_{max}^{2D}$ is approximately the same (Table 1).

**Figure 6:**
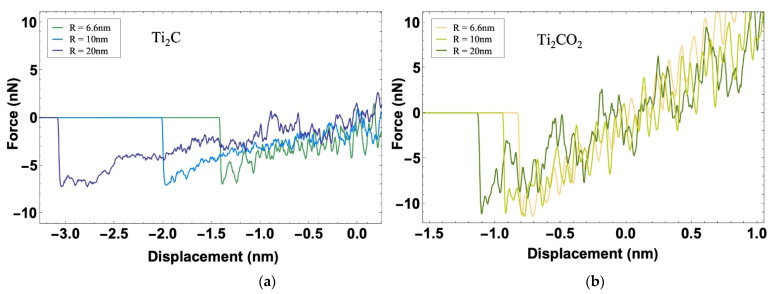
Force and displacement curves of (**a**) Ti_2_C and (**b**) Ti_2_CO_2_ of the withdrawal process using models with different hole radii (R = 6.6 nm, 10 nm, 20 nm).

According to the reported research, the tip radius is a model parameter that can affect the calculation of adhesion energy [30]. Here, we explore whether the hole radius also matters. The complete approach and withdraw process are carried out on the samples with different hole radii in the substrate (R = 6.6 nm, R = 10 nm, R = 20 nm). Figure 6 shows the force–displacement curves as the tip leaves the surface of MXene (Ti_2_C and Ti_2_CO_2_). The maximum height during the adhesion process of MXene flake vary significantly. The larger the radius of the hole, the higher the corresponding maximum height, especially for Ti_2_C, and the influence is much more significant. However, in the withdrawal process, there is almost no difference in the maximum value of adhesion force. Thus, our simulations reveal that the radius of the hole (R) is unrelated with the adhesion energy, but affects the deflection.

### 3.2. Friction Simulation

The friction was achieved by moving the horizontal virtual atom VL at a constant velocity of $3\;m\;s^{-1}$ (the spring constants $k_{x}=6\;N\;m^{-1}$) and causing the tip to slide across the surface. The normal load applied on the tip by the vertical spring ($k_{z}=0.15\;N\;m^{-1}$) is 0.6 nN. The lateral force and normal load are calculated by the following form:

(4) F=−kΔx

where k is a specified spring constant and Δx is the deformation of the spring.

**Figure 7:**
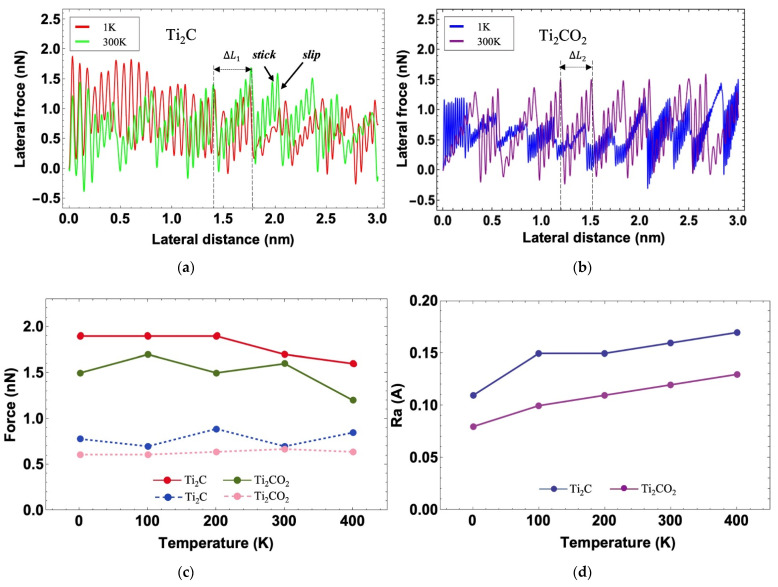
(**a**,**b**) Stick-slip behavior on Ti_2_C and Ti_2_CO_2_ under a normal force of 0.6 nN at 1 K and 300 K. (**c**) Maximum and average friction force of Ti_2_C and Ti_2_CO_2_ under a normal force of 0.6 nN at different temperatures. (**d**) Ra of Ti_2_C and Ti_2_CO_2_ at different temperatures.

The effects of the temperature on the stick-slip phenomenon of Ti_2_C and Ti_2_CO_2_ are shown in Figure 7a,b. It is evident that stick-slip phenomenon arises in a cyclic curve. The length of the stick is the same as the distance $\Delta L_{i}$ (shown in Figure 1a, $\Delta L_{1}=3.03\;Å$, $\Delta L_{2}=3.02\;Å$) between two nearest neighbor atoms in the X direction of MXenes. In contrast with Ti_2_C, we observed a more significant difference between the two curves of Ti_2_CO_2_, specifically the difference in minimal lateral force (Figure 7b). It indicates that the stick-slip phenomenon of Ti_2_CO_2_ is more sensitive to the temperature. To quantify the properties related to the friction of Ti_2_C and Ti_2_CO_2_ in different temperatures in our simulation, the maximum and average friction force were calculated and are displayed in Figure 7c. To further understand the causes of friction differences, we analyzed the surface roughness (*Ra*) (Figure 7d) by using formula 5:

(5) $$Ra=\frac{1}{n}\sum_{1}^{n}\left|z_{i}-\bar{z}\right|$$

where n is total number of the surface atoms on the MXene, $z_{i}$ is the z-coordinate value of a single atom and $\bar{z}$ is the average z-coordinate value of the surface atoms.

The Ra of Ti_2_C is always greater than Ti_2_CO_2_, which is the main reason why the average friction of Ti_2_C is higher than that of Ti_2_CO_2_. Additionally, the Ra of Ti_2_C and Ti_2_CO_2_ increases gradually with the increase in temperature. However, the average friction of Ti_2_C does not increase monotonously with the temperature, and there are some fluctuations. As a consequence, we can conclude that friction is not a simple linear correlation with roughness, and there are still many factors that affect friction that need to be explored in an atomic scale.

## 4. Conclusions

In this work, we developed two sets of models to study the adhesion and friction properties of Ti_2_C and Ti_2_CO_2_, with an emphasis on the effect of the temperature and O-functional group. We found that the temperature does affect the breaking point as well as the fracture morphology during indentation. Based on our atomistic simulation, we captured the “jump in” and “jump out” phenomenon and calculated the adhesion energy for the Ti_2_C and Ti_2_CO_2_ flakes. Based on our calculation, MXene containing oxide functional groups express a better adhesion property. Additionally, we noticed that the hole radius in the substrate may affect the adhesion behavior; thus, we conducted a series of simulation tests to study the effects of the radius of the hole in the substrate and compared our simulation results with the theoretical model in the literature. We found that the radius of the hole does have a great effect on the calculated elastic modulus, but almost no effect on adhesion energy. In the friction simulation, we evaluated the friction force and the surface roughness as well as the effect of the temperature. Through our research, we obtained a fundamental understanding of the surface properties of MXenes with and without the oxygen functional groups. Oxygen functional groups enhanced the elastic module $E^{2D}$ of MXenes and showed a greater performance in terms of adhesion. In addition, the knowledge of adhesion energy and friction obtained in our research are important for research and applications in many fields, especially lubrication [27], thin-film coatings [54], and composite design [15].

## Figures and Tables

**Table 1:** Elastic constants of Ti_2_C and Ti_2_CO_2_ calculated by plate elastic theory.

MXenes	$R\;\left(hole\right)\left(nm\right)$	$F_{f}\left(nN\right)$	$E^{2D}\;\left(N/m\right)$	$E\;\left(Gpa\right)$	$\sigma_{max}^{2D}\;\left(N/m\right)$
Ti_2_C	6.6	91	62	111	10.7
	10	105	63	113	11.5
	20	93	65	116	11.0
Average	-	96	63	113	11.1
Ti_2_CO_2_	6.6	55	93	155	10.1
	10	58	118	197	11.7
	20	52	133	222	11.8
Average	-	55	115	191	11.2

## Data Availability

The data presented in this study are available upon request from the corresponding author.
